# Advances in zearalenone-degrading enzymes research: characteristics, mining, improvement, and application

**DOI:** 10.1186/s40104-025-01281-y

**Published:** 2025-11-13

**Authors:** Yu Tang, Aimin Chen, Yongpeng Guo, Yanan Wang, Lihong Zhao

**Affiliations:** 1 State Key Laboratory of Animal Nutrition, Poultry Nutrition and Feed Technology Innovation Team, College of Animal Science andTechnology, China Agricultural University, Beijing, 100193 People’s Republic of China; 2 College of Animal Science and Technology, Henan Agricultural University, Zhengzhou, 450046 People’s Republic of China

**Keywords:** Feed, Feed ingredients, Mycotoxin, Zearalenone, Zearalenone-degrading enzymes

## Abstract

Zearalenone (ZEN) is a non-steroidal estrogenic mycotoxin that extensively contaminates feed and feed ingredients, posing a significant threat to animal health and food safety. Enzymatic degradation of ZEN is regarded as a promising strategy due to its high efficiency and safety. This review provides a comprehensive summary of recent advances in ZEN-degrading enzymes from a novel perspective, encompassing the types and catalytic mechanisms for characterizing ZEN-degrading enzymes, the methods for mining ZEN-degrading enzymes, the strategies for improving ZEN-degrading enzymes, and the applications of ZEN-degrading enzymes. The objective of this review is to offer a reliable reference framework for the enzymatic detoxification of ZEN in feed and feed ingredients, as well as to provide insights for mining other mycotoxin degrading enzyme in the future.

## Introduction

Mycotoxin contamination in crops is acknowledged as a significant threat to feed safety and animal health in global agriculture [1]. Zearalenone (ZEN), also known as F-2 mycotoxin, is one of the major non-steroidal estrogenic mycotoxins, mainly produced by *Fusarium* species, including *Fusarium graminearum*, *F. culmorum*, *F. cerealis*, *F. equiseti*, *F. crookwellense,* and *F. semitectum* [2]. Physically, ZEN is characterized as a white, crystalline, fat-soluble compound with a relatively high melting point of 164 to 165 °C. It is a phenolic resorcylic acid lactone mycotoxin with the molecular formula 6-(10-hydroxy-6-oxo-*trans*-1-undecenyl)-β-resorcylic acid lactone and the general formula C_18_H_22_O_5_. ZEN is soluble in chloroform, alcohols and some alkaline solutions [2, 3]. Its resistance to high temperatures, up to 150 °C, further contributes to its persistence in feed and feed ingredients [4]. Additionally, ZEN is recognized as one of the most prevalent mycotoxins in various animal feeds [5], and has also been detected in foods [6–8]. Furthermore, ZEN is defined as an estrogenic mycotoxin that interacts with estrogen receptors, eliciting estrogen-like effects that disrupt reproductive function of animals. Additionally, ZEN has been reported to elicit immunotoxin, genotoxin, and endocrine disrupting effects in animals [2, 3].

Given the health risks and economic losses associated with ZEN exposure in humans and animals, the control and removal of ZEN have garnered significant attention. It has been reported that managing agricultural practices, including field management, harvesting strategies, reasonable storage, and appropriate transportation can effectively control the contamination of *Fusarium* spp*.* for ZEN production [9–11]. For feed and feed ingredients already contaminated with ZEN, some physical, chemical, and biological methods have been reported for its removal [12, 13]. Physical detoxification methods include peeling, soaking, grinding, heat treatment, extrusion, irradiation, adsorption, extraction, cold plasma treatment, etc. Chemical detoxification methods include oxidation, alkali treatment, etc. Biological detoxification methods for the removal of ZEN mainly include reducing ZEN through silage or fermented feed [14, 15], degrading ZEN directly utilizing bacteria, fungi, and enzymes [12, 13], and breeding genetically modified crops with the capability to degrade ZEN [16, 17]. Among these methods, the enzymatic degradation of ZEN is particularly advantageous due to its high substrate specificity, mild reaction conditions, safe degradation products, operational simplicity, and cost-effectiveness, making it a current focal point of research [18].

This review provides a comprehensive summary of recent advances of ZEN-degrading enzymes from a novel perspective, encompassing the types and catalytic mechanisms for characterizing ZEN-degrading enzymes, the methods for mining ZEN-degrading enzymes, the strategies for improving ZEN-degrading enzymes, and the applications of ZEN-degrading enzymes. The objective of this review is to offer a reliable reference framework for enzymatic degradation of ZEN in feed and feed ingredients, and to provide insights for mining other mycotoxin degrading enzymes in the future.

## Characteristics of ZEN-degrading enzymes

The reported ZEN-degrading enzymes can be mainly categorized into four types based on their catalytic mechanisms for ZEN degradation: ZEN hydrolases, laccases, peroxidases, and other ZEN-degrading enzymes. These enzymes facilitate the degradation of ZEN, resulting in its hydrolysis or oxidation products.

### ZEN hydrolases

Among the ZEN-degrading enzymes, ZEN hydrolases have been the most extensively studied. ZHD101 is the most representative ZEN lactone hydrolase, and a series of ZEN lactone hydrolases reported subsequently are similar to it (the ZEN lactone hydrolases of ZHD101 types, ZHDs). Takahashi-Ando et al. [19] initially discovered ZHD101 from *Clonostachys rosea*. ZHD101 was homodimeric with a subunit molecular mass of 28.7 kDa, containing an intra-subunit disulphide bridge. It was capable of degrading ZEN under neutral and alkaline conditions (pH 9–10) and at physiological temperatures (37–42 °C). However, ZHD101 was rapidly inactivated at 50 °C, although it retained activity at 37 °C for one week [20]. ZHD101 had a core α/β-hydrolase domain, with a catalytic center composed of a S-H-E triad (S102-H242-E126) [21]. It has been characterized as cleaving the ester bond of the macrolactone ring in ZEN, and hydrolyze it to an unstable intermediate hydrolyzed zearalenone (HZEN), which spontaneously decarboxylates to yield decarboxylated hydrolyzed ZEN (DHZEN) [22]. Additionally, ZHD101 was capable of hydrolyzing zearalenols (ZOLs), resulting in non-toxic products. Nevertheless, its activity towards ZOLs was approximately 40% of that towards ZEN [20, 23]. Due to the extremely efficient ZEN degradation of ZHD101, its multiple homologs from different strains have been identified, including CbZHD [24], RmZHD (ZHD518) [25, 26], ZENG [27], and others [28–30]. These homologs exhibit similar properties and ZEN-degrading ability as ZHD101.

Recently, some novel ZEN hydrolases have been identified. Ji et al. [31] isolated and purified a hydrolase FSZ from *Aspergillus niger* ZEN-S-FS10, which efficiently degraded ZEN under acidic and neutral conditions. It was a novel ZEN hydrolase sharing less than 10% amino acid homology with ZHDs. Additionally, mass spectrometry analysis revealed a novel ZEN degradation product FSZ-P (C_18_H_26_O_4_). Shi et al. [32] identified another hydrolase ZENY from *Bacillus subtilis* YT-4 through genome BLAST, which shares 31% amino acid sequence identity with ZHD101. In addition, a new degradation product ZENY-C_18_H_24_O_5_ was determined through mass spectrometry. Similarly, some other novel ZEN hydrolases have been reported, including ZENH [33], ZTE138 [34], and ZENC [35]. Further research is required to elucidate their degradation mechanisms and to identify their degradation products. A comprehensive summary of these ZEN hydrolases was provided in Table 1.﻿

**Table 1:** The reported ZEN hydrolases currently

ZEN hydrolases	Calculated molecular mass	Optimal pH/temperature	K_cat_/K_m_	ZEN degradation products	Origins	Protein reference sequences in NCBI	Protein sequence similarity	References
GhZH	35.3 kDa	pH 7/42 °C	-/24.85 μg/mL	HZEN	*Gordonia hydrophobica* HAU421	XHP13120.1	75.5% with ZENH	[36]
ZH1-9 (Example of ZH9 only)	28.9 kDa	-	-	HZEN	*Exophiala mesophila*	XP_016229572.1	-	[37]
ZHRnZ	28.8 kDa	pH 9/45 °C	1.13 × 10^–4^ s^−1^·μg^−1^·mL^a^	-	*Rosellinia necatrix*	GAP90066.2	54.61% with ZHD101	[38]
ZHDAY3	29.3 kDa	pH 9.5/40 °C	-	-	*Exophiala aquamarina* CBS 119,918	XP_013255140.1	63% with ZHD101	[39]
ZENA	36.2 kDa	pH 8.2/38 °C	2.9 ± 0.1 s^–1^/0.34 ± 0.05 μmol/L	HZEN	*Rhodococcus erythropolis* PFA D8-1	AYJ13169.1	23% with ZHD101	[40]
ZENY	30.0 kDa	pH 8/37 °C	-	ZENY-C_18_H_24_O_5_	*Bacillus subtilis* YT-4	WQM43452.1	31% with ZHD101	[32]
ZHDR52 and ZHDP83	30.9 kDa and 28.7 kDa	pH 9.0/45 °C	-	-	*Gliocladium roseum*; *Gliocladium penicilloides* (Inconsistent with the authors, ZHDP83 is from *Clonostachys chloroleuca*)	Speculate as 5C7Y_A and CAI6078323.1	95.5% and 96.6% with ZHD101	[30]
ZHD607(ZHD11A)	28.7 kDa	pH 8/35 °C	12,262 s^−1^·M^−1a^	-	*Phialophora americana; Phialophora macrospora*	KIW70607.1	74% with ZHD518	[41, 42]
ZHD11D	29.4 kDa	pH 8/35 °C	86,857 s^−1^·M^−1^	-	*Phialophora attinorum*	XP_018004094.1	58.7% with ZHD518	[43]
ZENM	29.8 kDa	pH 9/60 °C	0.191 min^−1^·μM^−1a^	-	*Monosporascus* sp. GIB2	RYP08044.1	62% with ZHD101	[44]
ZENH	35.2 kDa	pH 7/55 °C	-/12.64 ± 0.16 μmol/L ^a^	ZENH-P1(C_20_H_18_O_5_)	*Aeromicrobium* sp. HA	WP_269305865.1	18.99% with ZHD101	[33]
ZHD11C	29.4 kDa	pH 7.0–8.0/45 °C	-	-	*Fonsecaea multimorphosa*	XP_016629930.1	73% with ZHD518	[45]
ZENR	36.1 kDa	pH 8–9/55 °C	-/21.14 ± 0.16 μmol/L^a^	HZEN	*Rhodococcus erythropolis*	WP_081559690.1	58% with ZENH	[46]
CLA; EXO; TRI	29.1 kDa; 29.3 kDa; 28.9 kDa	pH 7.0/40 °C; pH 9.0/40 °C; pH 9.5/40 °C	-	-	*Cladophialophora bantiana*; *Exophiala aquamarina*; *Trichoderma aggressivum*	XP_016613277.1; XP_013255140.1; AHG29544.1	61.22%, 62.88%, and 97.35% with ZHD101	[28]
ZHD_LD	28.9 kDa	pH 9.0/50 °C	-	-	*Exophiala spinifera*	XP_016234729.1	60.15% with ZHD101	[29]
ZHD11B	29.0 kDa	pH 8/44 °C	2,321 s^−1^·M^−1^	-	*Fonsecaea monophora*	XP_022506307.1	73.03% with ZHD518	[47]
FSZ	42.5 kDa	pH 2–8/28–38 °C	-/0.85 μg/mL^a^	FSZ-P (C_18_H_26_O_4_)	*Aspergillus niger* ZEN-S-FS10	GKZ72619.1	-	[31]
ZHD-P	28.9 kDa	pH 9/40 °C	-	-	*Trichoderma aggressivum*	AHG29544.1	97% with ZHD101	[48]
ZTE138	16.2 kDa	-	-	-	*Bacillus amyloliquefaciens* H6	WP_017417881.1	-	[34]
ZLHY-6	28.9 kDa	pH 8.5/37 °C	-	HZEN	-	AEV89971.1	98.3% with ZHD101	[49]
ZENG	28.8 kDa	pH 7/38 °C	-	-	*Gliocladium roseum* MA918	ALI16790.1	99% with ZHD101	[27]
RmZHD (ZHD518)	29.4 kDa	pH 8/40 °C	15,286 s^−1^·M^−1a^	HZEN	*Rhinocladiella mackenziei*	XP_013273750.1	65% with ZHD101	[25, 26, 47]
ZENC	32.1 kDa	pH 8/45 °C	-/38.63 ± 5.868 μmol/L^a^	-	*Neurospora crassa*	XP_963336.1	29% with ZHD101	[35]
CbZHD	29.1 kDa	pH 8/35 °C	-	-	*Cladophialophora bantiana*	XP_016613277.1	61% with ZHD101	[24]
ZHD101	28.7 kDa	pH 10.5/37–42 °C	1.48 × 10^4^ s^−1^·M^−1^	HZEN	*Clonostachys rosea* IFO 7,063	Q8NKB0.1	-	[19, 20]

^a^Measurements were taken at the optimal pH and temperature

**Fig. 1:**
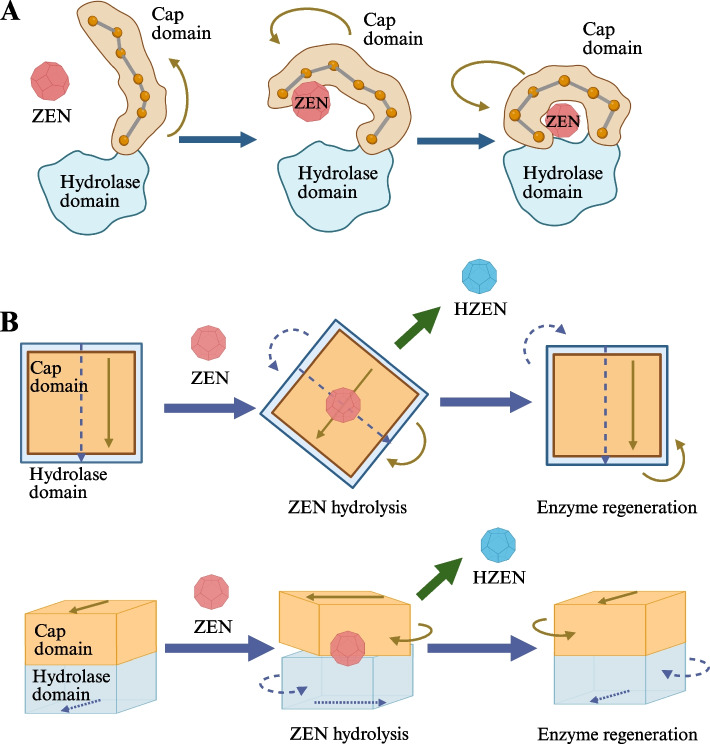
Dynamic interactions of ZHDs with ZEN. **A** Cap structure domains of ZHDs capture ZEN molecules and form a “sandwich” structure. **B** Occurrence of ZEN degradation reaction and enzyme regeneration in ZHDs

The degradation mechanism of ZHDs has been intensively verified and elucidated. Based on molecular docking simulations and single amino acid mutations, Wang et al. [25] identified RmZHD adopted core α/β-hydrolase domains with the active site situated at the C-terminal edge of the central β-sheet. The catalytic triad consists of S105-H243-E129, which was surrounded by α/β-hydrolase folds and helical cap domains. Zhou et al. [50] employed molecular dynamics simulations and quantum mechanics/molecular mechanics approaches to investigate the contributions of RmZHD residues in the degradation of ZEN. The energy barrier analysis of the reaction confirmed that the deprotonated nucleophile (S105) attacked the C1 atom of ZEN as the rate-determining step of the reaction. The amino acids E129, H243, S105, S106, and W185, along with substrate, formed the hydrogen bond network that stabilized the substrate and achieved the catalyzation of enzyme. Further, Hong et al. [51] analyzed the dynamic interaction of ZHDs with ZEN using molecular dynamics simulations. LIP, GMSRS, and SSGA were identified as conserved sequences in ZHDs using Boltzmann machine learning direct coupling analysis. Furthermore, molecular dynamics simulations elucidated the molecular motions at different stages of the process of ZEN hydrolysis by ZHDs. Briefly, prior to the docking stage, ZEN flowed around the ZHDs, while the cap structure domain of the protein unfolded akin to a tourniquet along the motile amino acid residue pathway. Once the ligand ZEN was captured, the cap domain constricted, facilitating the passage of ZEN between the cap domain and the hydrolase domain, ultimately entered the active center and formed a “sandwich” structure (Fig. 1A). Subsequently, the cap domain and the hydrolase domain rotated in opposite directions, initiating the hydrolysis reaction. Upon completion of the hydrolysis reaction, the two structural domains of the ZHDs reverted to their original positions, facilitating the release of product (Fig. 1B).

Overall, ZEN hydrolases are highly desired due to their excellent efficiency, safe products, and substrates specificity. Especially their exceptionally high ZEN catalytic efficiency. For instance, the K_cat_ and K_m_ values for ZEN hydrolase ZENA toward ZEN at 38 °C and pH 8.2 were 2.9 ± 0.1 s^−1^and 0.34 ± 0.05 μmol/L, respectively [40]. While the K_cat_ and K_m_ values for laccase CotA under similar conditions (at 37 °C, pH 8.0) toward ZEN were 0.11 s^−1^ and 90.43 μg/mL (approximately 284.2 μmol/L), respectively [52]. According to our calculations, the K_cat_/K_m_ values for ZENA and CotA are approximately 8.5 × 10^6^ s^−1^·M^−1^ and 3.87 × 10^2^ s^−1^·M^−1^, respectively. The former is about 22,000 times higher than the latter, indicating that under similar reaction conditions, the ZEN hydrolase exhibits superior degradation capacity for ZEN. In addition, ZEN hydrolases possess a lower molecular weight compared to laccases and peroxidases and are easier to heterologously express in a variety of engineered strains, both eukaryotic and prokaryotic. However, a significant limitation of ZEN hydrolases is their poor thermal stability, most of which inactivate within 5 min at 40–50 °C. Moreover, the majority of reported ZEN hydrolases degraded effectively ZEN only under neutral or alkaline conditions, demonstrating limited efficacy under acidic conditions. Therefore, future research directions for these enzymes may focus on improving their thermal stability, through molecular modification, immobilization, and the use of suitable carriers, as well as identifying novel hydrolases capable of maintaining activity under acidic conditions.

### Laccases for ZEN degradation

Laccases have been extensively reported for their ability to oxidatively degrade aromatic toxic compounds. Loi et al. [53] first reported that laccase Ery4 from *Pleurotus eryngii* degraded ZEN with redox mediator 2-azino-di-[3-ethylbenzo-thiazolin-sulphonate] (ABTS), syringaldehyde (SA), or 2,2,6,6-tetramethylpyperidyloxil. Wang et al. [54] reported that laccase CotA from *Bacillus subtilis* degraded ZEN with redox mediator methyl butyrate, caffeic acid, butyraldehyde, or vanillin. Qin et al. [55] reported that laccase StMCO from *Streptomyces thermocarboxydus* directly degraded ZEN without redox mediators, although the degradation efficiency was relatively low (8.58% ± 1.63%). Similarly, laccases Lac2 [56], rLac2 [57], etc. have been reported to degrade ZEN, albeit with the necessity of redox mediators.

Guo et al. [52] identified the first redox mediator-independent laccase CotA from *Bacillus licheniformis* ANSB821, demonstrating significant ZEN degradation activity with 96% degradation rate towards 10 μg/mL ZEN (12 h, pH 8, 37 °C). Subsequently, additional laccases from *Bacillus* were reported to degrade ZEN without redox mediators, including rCotA [58], CotA [59], BswLac [60], etc. Hao et al. [61] reported that laccase Lac-W from *Weizmannia coagulans* 36D1 degraded 60% of 4 μg/mL ZEN (24 h, pH 9, at room temperature) without redox mediators. Further, Jia et al. [62] reported ZEN degradation by Lac-W with redox mediators and identified the degradation product as 15-OH-ZEN using Ultra-High Performance Liquid Chromatography-Tandem Mass Spectrometry (UHPLC-MS/MS). In a separate study, Sun et al. [63] reported that laccases PpLac1 and AoLac2 from *Pleurotus pulmonarius* and *Aspergillus oryzae* respectively, directly degraded ZEN and exhibited outstanding acid resistance, functioning effectively at pH 2–6. A comprehensive summary of these ZEN-degrading laccases was provided in Table 2.

**Table 2:** The reported ZEN-degrading laccases currently

ZEN-degrading laccases	Calculated molecular mass	Optimal pH/temperature	ZEN degradation products	Origins	Protein reference sequences in NCBI	Redox mediators were essential to degrade ZEN	References
rLac2	56.7 kDa	-	-	*Cerrena unicolor*	QLF98712.1	Yes	[57]
BswLac	59.3 kDa	pH 8/55 °C	-	*Bacillus swezeyi*	WP_148958359.1	No	[60]
rCotA	58.5 kDa	pH 7/70 °C	-	*Bacillus subtilis* ZJ-2019–1	Speculate as WP_327828727.1	No	[58]
PpLac1; AoLac2	56.5 kDa; 53.2 kDa	pH 3–6/50 °C; pH 2–6/60 °C	15-OH-ZEN	*Pleurotus pulmonarius*; *Aspergillus oryzae*	KAF4592641.1; 6H5Y	No	[63]
Lac-W	59.7 kDa	-	15-OH-ZEN	*Weizmannia coagulans* 36D1	WP_014097300.1	No	[61, 62]
CotA	59.1 kDa	pH 8/75 °C	-	*Bacillus licheniformis* ANSB821	QAX90317.1	No	[52]
CotA	59.3 kDa	pH 9/80 °C	15-OH-ZEN and 13-OH-ZEN-quinone	*Bacillus licheniformis* ZOM-1	WP_020450420.1	No	[59]
StMCO	35.7 kDa	-	13-OH-ZEN-quinone	*Streptomyces thermocarboxydus*	WP_026243842.1	No	[55]
Lac2	56.6 kDa	pH 7/37 °C	-	*Pleurotus pulmonarius*	AAX40733.1	Yes	[56]
BsCotA	58.4 kDa	pH 5–10/50–80 °C	-	*Bacillus subtilis* 168	Speculate as WP_327828727.1	Yes	[54]
Ery4	58.1 kDa	-	-	*Pleurotus eryngii*	CAO79915.1	Yes	[53]

**Fig. 2:**
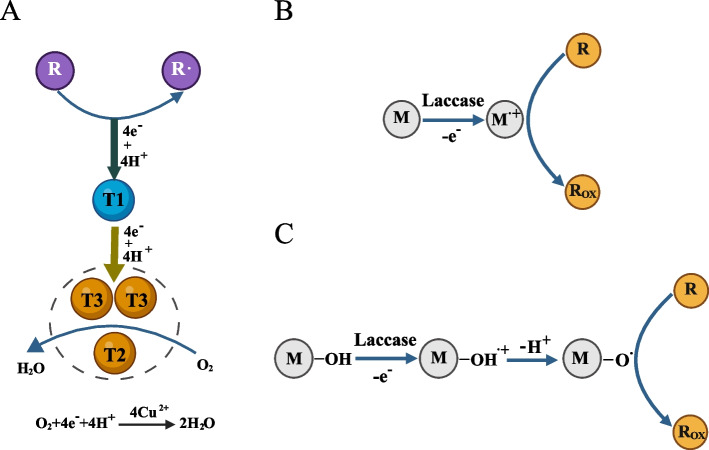
Oxidation reaction mechanism of laccases and laccase-mediated systems. **A** Oxidation reaction mechanism of laccases. **B** Electron transfer (ET) in laccase-mediated systems. **C** Hydrogen atom transfer (HAT) in laccase-mediated systems. R, substrates; Rox, oxidized substrates; M, redox mediator; T1, T2, T3, the four copper sites in laccases, classified as type 1 (T1), type 2 (T2), and binuclear type 3 (T3)

The mechanism of ZEN degradation by laccases has not been directly reported till now. Nevertheless, laccases, as substrate-broad oxidizing enzymes, have been extensively studied. Most laccases contain four copper sites, which are classified as type 1 (T1), type 2 (T2), and binuclear type 3 (T3), distinguished by their unique spectroscopic characteristics. Specifically, the absorption bands for T1-Cu and T3-Cu are observed at 600 nm and 330 nm, respectively, while T2-Cu does not exhibit an observable absorption band [64, 65]. The oxidation of substrates occurs in the vicinity of T1 site, where an electron is extracted from the substrate and accepted by the T1-Cu at the active center, yielding a substrate radical cation. Subsequently, this electron is transferred from the T1-Cu through the Cyc-His pathway to the T2/T3 Cu cluster, which utilizes the electron to reduce O_2_ to H_2_O, thereby regenerating the enzyme [66, 67] (Fig. 2A).

Redox mediators are small molecules that function as electron carriers. Upon oxidation by laccases, these mediators exit the active site and diffuse into the solvent to oxidize substrates that are structurally unable to access the active site directly. Alternatively, the redox mediators can oxidize the substrates through mechanisms different from that of laccases, thereby indirectly broadening the range of substrates amenable to oxidation by laccases [68, 69]. Laccases employ two primary mechanisms to oxidize substrates via redox mediators: electron transfer (ET) and hydrogen atom transfer (HAT). In ET, laccase extracts an electron from the redox mediator, transforming the redox mediator into a radical cation that subsequently oxidizes the substrate, as exemplified by ABTS-mediated substrate oxidation (Fig. 2B). In HAT, laccase extracts an electron from the redox mediator, next the redox mediator removes a H atom on its own (deprotonation), resulting in an uncharged radical that further oxidizes the substrate [68, 69]. Redox mediators with N–OH structural characteristics, such as 1-hydroxybenzotriazole, violuric acid, 3-hydroxyanthranilic acid, and N-hydroxy-phthalimide, preferentially follow the HAT pathway [65] (Fig. 2C). Similarly, natural redox mediators (including acetosyringone, syringaldehyde, vanillin, acetovanilline, ferulic acid, p-coumaric acid, etc.) usually follow the HAT oxidation mechanism [65].

Laccases, particularly those from *Bacillus*, exhibit better stability and are able to withstand high temperatures and long-term storage. They possess the ability to oxidatively degrade a wide range of toxic compounds and mycotoxins, including ZEN, bisphenol A, aflatoxin B_1_ (AFB_1_), *Alternaria* toxin alternariol, etc. [59, 70]. Nonetheless, their catalytic efficiency towards ZEN remains suboptimal, compared with ZEN hydrolases. The broad substrate range also enables them to oxidize some beneficial compounds, potentially limiting their application. In addition, they demonstrate limited efficacy in degrading ZEN under acidic conditions. Therefore, the development of more efficient laccases for ZEN degradation under acidic conditions may be a future research direction.

### Peroxidases for ZEN degradation

Peroxidases are capable of catalyzing the oxidation of many organic and inorganic substrates utilizing H_2_O_2_ as a mediator, and have been widely studied in the degradation of lignin, dyes, antibiotics, pesticides, and many other contaminants [71]. Many commercially available peroxidases, such as horseradish peroxidase, and those extracted from soybean bran, soybean hulls, and rice bran, have been reported to degrade ZEN using H_2_O_2_ [72–74]. For the heterologously expressed peroxidases, their degradations toward ZEN often required redox mediators. Wang et al. [75] evaluated eight manganese peroxidases (MnPs) from lignocellulose-degrading fungi, all of which successfully degraded the four major mycotoxins AFB_1_, ZEN, deoxynivalenol (DON), and fumonisin B_1_ with Mn^2+^, H_2_O_2_, and dicarboxylic acid malonate. Qin et al. [76] reported that the dye decolorizing peroxidase BsDyP from *Bacillus subtilis* SCK6 degraded AFB_1_, ZEN, and DON with Mn^2+^, H_2_O_2_, and dicarboxylic acid. Similarly, many peroxidases degraded ZEN required redox mediators more than H_2_O_2_, such as: RhDypB [77], BsDyP [78], BaDyP [79], etc. A comprehensive summary of these ZEN-degrading peroxidases was provided in Table 3.

**Table 3:** The reported ZEN-degrading peroxidases currently

ZEN-degrading peroxidases	Calculated molecular mass	Optimal pH/temperature	ZEN degradation products	Origins	Protein reference sequences in NCBI	Notes	References
BaDyP	53.0 kDa	-	15-OH-ZEN and HZEN	*Bjerkandera adusta*	CDN40127.1	Dye-decolorizing peroxidase; contained heme; Mn^2+^ or 1-HBT, H_2_O_2_ were necessary	[79]
PhcMnp; IrlMnp	38.6 kDa; 37.2 kDa	pH 4.5/30–50 °C; pH 4.5/30 °C	-	*Phanerochaete chrysosporium*; *Irpex lacteus*	AAA33746.1; AQT03613.1	Manganese peroxidase; contained heme; Mn^2+^, H_2_O_2_, and dicarboxylic acid malonate were necessary	[80]
PoDyP4	57.2 kDa	pH 6/40 °C	15-OH-ZEN, 14-OH-ZEN-quinone, 13,15-OH-ZEN, ZEN dimer, and 15-OH-ZEN dimer	*Pleurotus ostreatus*	Speculate as KAJ8690841.1	Dye-decolorizing peroxidase; contained heme; H_2_O_2_ was necessary	[81]
Soybean hull peroxidase	38.1 kDa	pH 8/57 °C	13-OH-ZEN and 13-O-ZEN-quinone	*Glycine max*	AAL77517.1	Contains heme; H_2_O_2_ was necessary	[74]
RhDypB	37.8 kDa	-	15-OH-ZEN	*Rhodococcus jostii*	AYJ72200.1	Dye-decolorizing peroxidase; contained heme; Mn^2+^, H_2_O_2_, and dicarboxylic acid were necessary	[77]
BsDyP	45.7 kDa	pH 8/42 °C	ZEN-11,12-oxide	*Bacillus subtilis *168	WP_003243445.1	Dye-decolorizing peroxidase; contained heme; Mn^2+^, H_2_O_2_, and dicarboxylic acid were necessary	[78]
StDyP	45.5 kDa	-	15-OH-ZEN and 13-OH-ZEN-quinone	*Streptomyces thermocarboxydus* 41291	WP_019525974.1	Mn^2+^ or 1-HBT, H_2_O_2_ were necessary	[82]
BsDyP	45.7 kDa	-	15-OH-ZEN	*Bacillus subtilis *SCK6	WP_124059398.1	Dye-decolorizing peroxidase; contained heme; Mn^2+^, H_2_O_2_, and dicarboxylic acid are necessary	[76]
Ase	26.9 kDa	pH 9/50 °C	-	*Acinetobacter* sp. SM04	QAB35658.1	Contained heme and degraded ZEN without H_2_O_2_	[83]
Commercial peroxidase; peroxidases from soybean bran and rice bran	-	pH 7–8/30 °C	2,4-O-dimethyl-ZEN	*Armoracia rusticana*; *Glycine max*; *Oryza sativa L*	-	H_2_O_2_ was necessary	[72, 73]
*Il*MnP1/2/4/5/6; *Nf*MnP; *Pc*MnP1; *Cs*MnP (Example of *Il*MnP1 only)	40.1 kDa	-	-	*Irpex lacteus* CD2	AAA33746.1	Manganese peroxidase; Mn^2+^, H_2_O_2_, and dicarboxylic acid malonate were necessary	[75]
Prx	20.7 kDa	pH 9/70 °C	-	*Acinetobacter* sp. SM04	MBP2602639.1	H_2_O_2_ was necessary	[84]

**Fig. 3:**
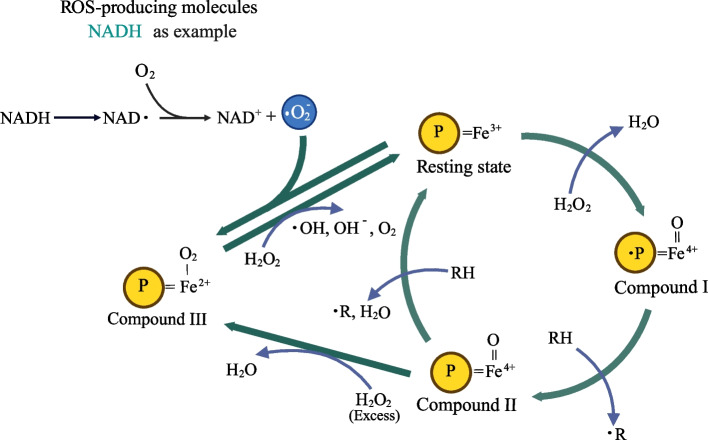
Oxidation reaction mechanism of peroxidases for decomposing H_2_O_2_. R, substrates; P, porphyrin

The mechanism of ZEN degradation by peroxidases has not been reported. Nevertheless, the mechanism of peroxidases to degrade other substrates is referable, which may suggest a possible mechanism for ZEN degradation. Peroxidases utilize a common catalytic mechanism for decomposing H_2_O_2_, which has been elucidated in class III peroxidases (from horseradish, barley, soybean, etc.). This process, known as the Poulos-Kraut mechanism, involves of three irreversible steps within a classic two-electron redox reaction [85, 86] (Fig. 3): (1) H_2_O_2_ interacts with the iron atom (Fe^3+^) of the heme group, facilitating the transfer of a proton from the O1 atom of H_2_O_2_ to the O2 atom. This results in the cleavage of the O–O bond, yielding a water molecule and an oxyferroporphyrin cation radical (compound I). (2) Subsequently, the O1 atom of compound I accepts two electrons from the enzyme. One electron is transferred from the iron atom, converting Fe^3+^ to Fe^4+^ and forming compound II (Fe^4+^) with an oxygen-iron (Fe = O) center. The second electron is transferred from the porphyrin ring, yielding a porphyrin π-cation radical, which subsequently acquires an electron from the electron-donor substrate, leading to the oxidation of the substrate. (3) Lastly, compound II (Fe^4+^) accepts another electron from the second substrate molecule, resulting in oxidation of the substrate and the formation of another H_2_O molecule. This process also restores the enzyme to its initial state (Fe^3+^). Moreover, some molecules that yield reactive oxygen species (ROS), such as thiols [RSH], salicylic acid, or NAD(P)H, derived radicals (i.e., [RSSR]^− •^, SA^•^ and NAD(P)^•^) react with O_2_ to yield superoxide anion radicals (O_2_^− •^), which can reduce peroxidase in its resting state to form compound III. In the presence of H_2_O_2_, compound III in the Fe^2+^-O_2_ state yields an OH^•^ through the Fenton-type reaction (Fe^2+^-O_2_ + H_2_O_2_ → Fe^3+^ + OH^•^ + O_2_) [87]. This mechanism may explain the hydroxylation modifications observed in the ZEN degradation products of peroxidases.

Peroxidases are extensively available, including commercial products and extracts from soybean hulls, wheat bran, etc. These enzymes exhibit a broad substrate specificity, enabling the degradation of various mycotoxins and contaminants. However, they also have the capacity to oxidize beneficial compounds. Furthermore, the dependence on H_2_O_2_ is a significant limitation for their application in the feed industry. Consequently, the effective utilization of peroxidases for mycotoxin degradation may require special application strategies that have yet to be established.

### Other ZEN-degrading enzymes

Some enzymes reported to degrade ZEN have not been categorized within the previously mentioned types, suggesting potential directions for identifying novel ZEN-degrading enzymes. Cheng et al. [88] identified a lactonase AttM from *Bacillus megaterium* HNGD-A6 through genome BLAST, which degraded 67.82% of ZEN at pH 8.5, 80 °C. The lactonase AttM was annotated as a multicopper oxidase, exhibiting 31.78% similarity with the N-acyl endonuclease of *Bacillus*. Similarly, another oxidase Oxa [89] from *Acinetobacter sp.* SM04 has also been reported to degrade ZEN. Tang et al. [83] reported a heme-containing oxidase Ase from *Acinetobacter* sp. SM04, which exhibited peroxidase activity and degraded ZEN without H_2_O_2_. Adegoke et al. [90] reported that two pore proteins Peroxiredoxin and Porin from *Acinetobacter nosocomialis* Y1 transformed ZEN to β‐zearalanol (β-ZAL). In particular, Porin shared 91% sequence similarity with peroxidase Ase [83], although the authors did not test the addition of H_2_O_2_ to assess peroxidase activity. A comprehensive summary of these other ZEN-degrading enzymes was provided in Table 4.

**Table 4:** Other ZEN-degrading enzymes not categorized

ZEN-degrading enzymes	Calculated molecular mass	Optimal pH/temperature	Origins	Protein reference sequences in NCBI	Notes	References
Oxidase Oxa	27.6 kDa	pH 9/60 °C	*Acinetobacter* sp. SM04	WP_005078653.1	The engineered strain *L. acidophilus* pMG-Oxa degraded 42.95% of ZEN (20 μg/mL) in 12 h	[89]
Lactonase AttM	30.1 kDa	-	*Bacillus megaterium* HNGD-A6	WQM43578.1	Multicopper oxidase	[88]
Peroxiredoxin and Porin	20.8 kDa; 27.8 kDa	pH 9/60 °C	*Acinetobacter nosocomialis* Y1	WP_002049519.1; WP_002053751.1	The ZEN degradation products is β-ZAL	[90]

Moreover, mimetic enzymes are artificial synthetic constructs designed by integrating the structural characteristics of natural enzymes and emulating their catalytic mechanisms. Chen et al. [91] drew inspiration from lactone hydrolases to develop a peptidyl enzyme mimetic material for the ZEN degradation. It was achieved by combining a S/H/E catalytic triad with a pro-hydrophobic self-assembling sequence and an oxygen anion cavity site, resulting in the ZEN degradation of 74.14% within 30 h (ZEN 1 μg/mL, at 35 °C, pH 8). Currently, the application of mimetic enzymes technology have been reported in laccase [92] and peroxidase [93], suggesting a promising potential for ZEN degradation.

In summary, no inherent superiority exists among the various types of ZEN-degrading enzymes.It is advisable to assign them separate roles and specialize their development based on specific practical application scenarios. From our perspective, ZEN hydrolase appears to be the most promising due to its exceptional catalytic efficiency and specificity toward ZEN. However, all the reported natural ZEN-degrading enzymes need further modification to meet practical industrial demands. For ZEN hydrolases, they need to be enhanced in thermal stability and acid tolerance; for laccases, they need to be enhanced in catalytic efficiency and substrate specificity toward ZEN; and for peroxidases, their dependence on hydrogen peroxide needs to be adequately addressed.

### Enzymatic degradation products of ZEN

Currently, studies on the enzymatic degradation products of ZEN remains limited, with some products having been only superficially identified. Further investigation into the physicochemical properties and bioactivities of these products is necessary.

#### Enzymatic hydrolysis products

HZEN and DHZEN are products of ZEN lactone hydrolysis yielded by ZHDs. Vekiru et al. [22] identified the primary reaction product HZEN using liquid chromatography tandem mass spectrometry. They subsequently purified it through preparative HPLC, and confirmed its postulated structure, (E)-2,4-dihydroxy-6-(10-hydroxy-6-oxo-1-undecen-1-yl) benzoic acid, using nuclear magnetic resonance techniques. Further, the spontaneous decarboxylation of DHZEN, ((E)-1-(3,5-dihydroxy-phenyl)-10-hydroxy-1-undecen-6-one), was also identified. The mass spectrometry characteristic for HZEN and DHZEN are presented in Table 5.

**Table 5:** ZEN and its enzymatic degradation products

Compounds	Products of chemical reaction	Molecular formula/Exact mass	[M-H]^−^ ions (*m/z*, ESI (-))	Fragments (*m/z*, ESI (-))	Structures	References
ZEN	-	C_18_H_22_O_5_/318.1467	317.1451	149.1, 175.1, 273.5, 299.4		[94]
HZEN	Hydrolysis product	C_18_H_24_O_6_/336.1573	335.1572	107.1, 149.1, 161.1, 291.2		[36]
DHZEN	Hydrolysis product	C_17_H_24_O_4_/292.1675	291.1648	123.1, 149.1, 161.1		[46]
15-OH-ZEN	Oxidation product	C_18_H_22_O_6_/334.1416	333.134	175.1, 203.1, 289.2, 315.4		[94]
13-OH-ZEN-quinone	Oxidation product	C_18_H_20_O_6_/332.126	331.1186	202.1, 287.2, 303.1, 312.2		[94]

The current understanding of the toxicity of HZEN and DHZEN is largely derived from studies on the mixed products of enzyme-degraded ZEN. Fruhauf et al. [95] demonstrated that HZEN and DHZEN did not induce an estrogenic response, as confirmed through female piglets, MCF-7 cells, and estrogen-sensitive yeasts. These metabolites were at least 50–10,000-fold less estrogenic than ZEN in vitro. Pierron et al. [96] reported that HZEN (40 μmol/L) had no effect on the proliferation of porcine peripheral blood mononuclear cell, total B cells, and B cell subsets (CD21^+^, IgM^+^, and IgG^+^ B cells), nor on the production of B cell antibodies (IgM, IgG, and IgA). Tassis et al. [97] reported that HZEN (62.8 μmol/L) showed no significant effect on the kinetics, morphology, and viability of boar semen in vitro for 4 h. In some other cell experiments, the ZEN degradation products were verified not to have effects on the activity of the cells [36, 79]. However, these reports are still superficial, and more systematic studies on the toxicity of HZEN and DHZEN are urgently needed.

In addition, some hydrolyzed products of ZEN-degrading enzymes have been reported but not completely identified, including ZENY-C_18_H_24_O_5_ [32], FSZ-P [31], ZENH-P1 [33], etc.

#### Enzymatic oxidation products of ZEN

15-OH-ZEN and 13-OH-ZEN-quinone are among the most reported ZEN oxidation products catalyzed by laccases and peroxidases. Qin et al. identified 13-OH-ZEN-quinone ((S,E)-14-hydroxy-3-methyl-3,4,5,6,9,10-hexahydro-1H-benzo[c][1]oxacyclotetradecine-1,7,13,16(8H)-tetraone) and 15-OH-ZEN ((S,E)-14,15,16-trihydroxy-3-methyl-3,4,5,6,9,10-hexahydro-1H-benzo[c][1]oxacyclotetradecine-1,7(8H)-dione) as ZEN oxidation products by laccase StMCO [55], peroxidase StDyP [82], or peroxidase BsDyP [76]. The mass spectrometry characteristic for 15-OH-ZEN and 13-OH-ZEN-quinone are presented in Table 5.

Systematic research on the toxicity of these ZEN oxidation products remains absent. In vitro cellular experiments have been conducted to simply assess the toxicity of mixed products resulting from the enzymatic oxidation of ZEN, including 15-OH-ZEN [59, 79, 81], 13-OH-ZEN [74], 13-OH-ZEN-quinone [55, 59, 74], and ZEN-11,12-oxide [78], which were all considered less toxic than ZEN. However, toxicity studies of ZEN oxidation products still lack animal experimental data.

## Methods for mining ZEN-degrading enzymes

The efficient and reliable methods for mining ZEN-degrading enzymes are crucial. There are several validated methods for mining these enzymes as follows.

### Methods based on enzyme separation and purification from wild microbes

The initial method for screening novel ZEN-degrading enzymes from wild microbes was through protein isolation and purification techniques (e.g., salt precipitation and column chromatography) [19]. Using this method, Takahashi-Ando et al. [19] successfully identified the first ZEN-degrading enzyme, ZHD101. Concretely, ZEN-degrading strain *C. rosea* IFO 7063 was cultured, and its mycelium was collected, pulverized, and centrifuged. ZHD101 present in the centrifuged supernatant was precipitated using ammonium sulfate, concentrated by dialysis, and subsequently purified by ion exchange chromatography and gel filtration chromatography. The amino acid sequence of the purified ZHD101 was determined by protein sequencing. Corresponding cDNAs were amplified and cloned using rapid amplification of cDNA ends technology, followed by sequencing. The assembled nucleotide sequence of cDNA revealed an open reading frame encoding ZHD101. Finally, ZHD101 was heterologously expressed in *E. coli* and verified. Similarly, ZEN-degrading enzymes FSZ [31], Ase [83], Oxa [89], etc. were obtained and verified by this method.

Nevertheless, this method usually necessitates long experiment cycles, including microbial cultivation, enzyme extraction, and subsequent verification. Complex extraction and purification steps are also detrimental to the stability of certain enzymes, thereby necessitating the use of mild buffer systems and low temperatures conditions to maintain enzymatic activity. In addition, the enzymes extracted from wild microbes inherently retain their natural activities, due to the preservation of native splicing, post-translational modifications, and appropriate prosthetic groups, which may differ from those expressed in heterologous systems.

### Methods based on similarity and validation of enzymes

A method commonly used for the identification of potential novel ZEN-degrading enzymes involves assessing their similarity with previously reported ZEN-degrading enzymes. This process is achieved by constructing expression vectors based on the BLAST analysis of reported ZEN-degrading enzymes in databases, followed by validation through heterologous expression. For instance, Hui et al. [24] selected a potential ZEN hydrolase cbZHD, which shared 61% protein sequence identity with ZHD101, by BLAST analysis within National Center for Biotechnology Information database (https://www.ncbi.nlm.nih.gov/). The cbZHD was subsequently expressed heterologously and verified to possess ZEN-degrading activity. Similarly, ZEN-degrading enzymes RmZHD (ZHD518) [25], CotA [52], RhDypB [77], etc. were identified by this method.

This method based on similarity is relatively straightforward and efficient. However, it tends to yield enzymes that are less innovative and possess properties similar to those previously reported. Consequently, it primarily facilitates the exploration of known enzymes, presenting challenges in the discovery of new degradation pathways or novel enzymes with distinct characteristics.

### Methods based on omics and validation

In recent years, omics have emerged as valuable tools for the mining of ZEN-degrading enzymes. Xu et al. [34] employed transcriptome sequencing to analyze the overexpressed transcripts of *Bacillus amyloliquefaciens* H6 strain incubated with ZEN. As a result, the YBGC/FADM family acyl-coenzyme A thioesterase ZTE138 was predicted to be the key protein responsible for ZEN degradation, which was verified to degrade 59.79% of ZEN (1 μg/mL) at 37 °C, after 72 h incubation. Hu et al. [33] measured the fermentation supernatant of *Aeromicrobium* sp. HA strain by comparative proteomics, and six enzymes from the reported family of ZEN-degrading enzymes were selected and heterologously expressed in *E coli*. Among these, an α/β-fold hydrolase ZENH was validated as a novel ZEN-degrading enzyme. Similarly, ZEN-degrading enzymes ZENY [32], GhZH [36], AttM [88], etc. were identified by this method.

Omics-based validation approaches are emerging methods for mining the novel ZEN-degrading enzymes that are more differentiated than those previously reported. Compared to isolation and purification, this method is less time-consuming and is not limited by enzyme stability. Nonetheless, this method is contingent upon the quality and comprehensiveness of existing databases, as well as the subjective choice made by researchers. It is desirable to adopt a comparative omics method to compare the microbial responses to ZEN under varying culture conditions (e.g., different media, oxygen levels and culture durations), thereby reducing the number of potential degrading enzymes that require verification.

### Methods based on artificial intelligence

In recent years, artificial intelligence methods are increasingly being employed in enzyme studies. Zhang et al. [37] proposed a robust model for predicting enzyme substrate promiscuity based on positive unlabeled learning. Based on the predictions of this model, ten potential ZEN hydrolases and ten potential ochratoxin A (OTA) hydrolases were selected and verified. The maximum sequence identity of the selected sequences relative to the training set was 32%–40%, indicating the novelty of the newly identified enzymes. Upon expression in a rapid cell-free protein expression system, nine enzymes exhibited significant catalytic activity towards ZEN and six enzymes exhibited significant catalytic activity towards OTA.

The revolutionary impact of big data and artificial intelligence on enzyme engineering is undeniable, and the identification and de novo design of mycotoxin-degrading enzymes through these methods represent a foreseeable opportunity of the current era. This field is still in its infancy, while such methods are being applied to identify and design de novo various enzymes, such as peroxidases [98], which have the potential to degrade ZEN.

### Other methods

Some other methods have been reported to mine ZEN-degrading enzymes. One such method involves a validation approach based on the segmented expression of ZEN-degrading bacterial genomes. Fruhauf et al. [40] identified a ZEN hydrolase ZENA from *Rhodococcus erythropolis* PFA D8-1 by this method. Briefly, they segmentally transferred the genomic library of *R. erythropolis* PFA D8-1 (degrade ZEN) into *R. erythropolis* PR4 (not degrade ZEN), and verified whether the transgenic PR4 strain acquired the ZEN-degrading ability. Ultimately, an open reading frame encoding the ZEN hydrolase ZENA was identified and expressed in *E. coli*. However, this method is complex and time-consuming, and the protein sequence of ZENA is unfortunately identical to ZENR obtained through genome BLAST by Hu et al. [46]. In a similar study, Altalhi et al. [99] segmented the 120 kb pZEA-1 plasmid gene from *Pseudomonas putida* ZEA-1 and expressed them in* E. coli*. A 5.5 kb fragment containing a ZEN-degrading enzyme was finally obtained. Nevertheless, the specific enzyme responsible for ZEN degradation failed to be conclusively identified.

In summary, each method for identifying ZEN-degrading enzymes possesses distinct advantages, and no single approach can be deemed superior. We suggest that future endeavors in mining ZEN-degrading enzymes should adopt a synergistic strategy of various methods, selecting and combining different methods based on specific instances. For instance, when mining ZEN-degrading enzymes from a readily accessible and cultivable strain, it is advisable to combine enzyme isolation and purification with transcriptomic methods. Conversely, when dealing with a strain that is challenging to obtain or culture, a combination of similarity-based BLAST and genomic methods is recommended.

## Strategies for improving ZEN-degrading enzymes

Various molecular modification strategies have been employed to enhance the stability and catalytic efficiency of ZEN-degrading enzymes. Additionally, advancements have been achieved in optimizing the expression systems of these enzymes to improve their expression efficiency and adapt to potential application scenarios.

### Molecular modifications of enzyme based on bioinformatics tools

In recent years, with the growing understanding of enzyme molecular structure and function, molecular modeling and docking tools have been developed and applied to significantly facilitate enzyme improvement. Some emerging molecular biology simulation tools (e.g., Discovery studio, PyMol and YASARA) along with homology modeling tools (e.g., SWISS-MODEL, I-TASSER, trRosetta Suite and AlphaFold) have been developed and validated, which can give provide extensive simulations of enzyme–substrate interactions, enabling the identification of key amino acids for targeted mutagenesis.

#### Modifications of enzymatic activity centers

Single amino acid mutations based on rational design are frequently employed to modify one or a few amino acids in the active center of enzymes in order to alter substrate binding affinity or improve the catalytic efficiency. For instance, Xu et al. [23] improved the degradation of α-ZOL by molecular modification of ZHD101 based on crystal diffraction and molecular docking. The aliphatic C8 of α-ZOL might repel the hydrophilic H242 side-chain imidazole with a hydrophobic force, disrupting the S102-H242-E126 hydrogen bond network. Therefore, the V153H mutant was designed to stabilize the lactone ring via a hydrogen bond between the H153 side chain and the C6-OH of α-ZOL, allowing H242 to rotate back to its correct orientation and thereby restoring the functional catalytic triad. Finally, the enzymatic activity of V153H towards α-ZOL was increased by 3.7-fold while its activity towards ZEN remained unchanged. This mechanism was further elucidated by Liu et al. [100]. Similarly, ZEN-degrading enzymes ZHD101-M2/M9 [101], RmZHD (ZHD518)-N156H [25]/Y160A [26], ZHD607-I160Y [41], etc. were molecularly modified through analogous methods. A comprehensive summary of these mutants was presented in Table 6.

**Table 6:** Molecular modifications of ZEN-degrading enzymes

ZEN-degrading enzymes	Modifications in enzymatic activity centers	Functions	References
ZHRnZ-E122R	No	Enhanced thermal stability and catalytic activity	[38]
ZHDAY3-N153H	Yes	Enhanced catalytic activity toward α-ZOL, α-ZAL, and β-ZAL derivatives	[39]
ZHD11A-I160Y-G242S	No	Enhanced thermal stability and specific catalytic activity of ZHD11A through Firefrot analysis (https://loschmidt.chemi.muni.cz/fireprotweb/)	[42]
ZENY-N5V/NΔ11	No	Enhanced thermal stability through the change at the N-terminus of ZENY	[32]
ZHD101-T229C/D170C	No	Enhanced thermal stability of ZHD101 through DbD2 analysis (http://cptweb.cpt.wayne.edu/DbD2/)	[102]
ZHD101-M1/M2	No	Enhanced thermal stability and pH stability	[103]
ZENM-G163S	Yes	Preferring α-ZOL as its optimum substrate	[44]
ZHD518-T6V and ZHD101-T6V/R52T	No	Enhanced thermal stability through the change at the N-terminus of ZHDs	[45]
ZENG-S162P/S220R	No	The half-life of the mutant at 55 °C was enhanced by 36.8-fold, and the melting temperature was enhanced by 8.2 °C	[104]
ZHD607-ZHDM1/I160Y	No	Enhanced catalytic activity by 2.9- and 3.4-fold	[41]
ZHD101-M2/M8/M9	Yes	M2(D157K) and M9(E171K) enhanced the catalytic activity of ZHD101 under acidic conditions; M8(D133K) and M9(E171K) enhanced the turnover numbers by 2.73- and 2.06-fold	[101]
ZENG-H143F/S143F, H143L/S143L, and H143I/S143I	No	Enhanced thermal stability	[27]
ZHD518 (RmZHD)-N156H	Yes	Enhanced catalytic activity toward ZEN, α-ZAL and β-ZAL, reduced catalytic activity towards β-ZOL	[25]
ZHD518 (RmZHD)-Y160A	Yes	Enhanced the α-ZOL catalytic activity by more than 70%	[26]
ZHD101-V153H	Yes	Maintained catalytic activity towards ZEN and enhanced specific catalytic activity towards α-ZOL by 3.7-fold	[23]

Nevertheless, this method requires a highly accurate model of the enzyme's molecular structure, such as that provided by X-ray diffraction data of enzymes and substrates, which is often unavailable for newly discovered enzymes. In addition, modifications to the active center of enzymes tend to have a more significant effect on substrate binding or catalytic efficiency, while exerting a comparatively less effect on the stability, solubility, or pH suitability of enzymes.

#### Modifications of non-enzymatic activity centers

The rational design to non-enzymatic activity centers of enzymes using molecular biology simulation software is an effective method to improve enzyme stability or pH suitability. In a recent report, Ding et al. [102] designed the mutant ZHD101-T229C/D170C based on the crystal structure of ZHD101, achieving a significant improvement in thermal stability without compromising catalytic efficiency. Specifically, intermolecular disulfide bonds were introduced to link the two ZHD101 monomers, and B-factor analysis was employed to identify mutation sites with lower energy, all located outside the active center of ZHD101. Compared with the wild type, ZHD101-T229C/D170C exhibited a 7 °C increase in thermal half-inactivation temperature, a 200% increase in half-life at 50 °C, and an 18.1 °C increase in melting temperature. In a separate report, Wang et al. [45] found that the hydrophobicity of the N-terminus of ZHD11C influenced its thermal stability and induced conformational changes in the distal structural domains. Further, the mutants ZHD518-T6V and ZHD101-T6V/R52T were rationally designed using I-TASSER, and verified to have higher thermal stability. Similarly, ZEN-degrading enzymes ZENG-S162P/S220R [104], ZHD11A-I160Y-G242S [42], ZHD607-ZHDM1 [41], etc. were molecularly modified through analogous methods. A comprehensive summary of these mutants was presented in Table 6.

However, some studies [101] have indicated that the effects of multiple amino acid mutations on enzymes within rational design were often non-additive, which might be attributed to the heterogeneous nature of protein structures, and many molecular biology simulation tools currently lack the capability to accommodate such complexities.

In recent reports, some novel predictive methods concerning the energy and charge of amino acid residues have been used for enzyme modification. In instance, Liu et al. [38] designed the potential mutation site E122 by calculating the Root Mean Square Fluctuation (RMSF) values of individual amino acid residues in the ZEN hydrolase ZHRnZ. Through single-amino acid saturation mutation, the mutant ZHRnZ-E122R was verified to have higher thermal stability and catalytic activity. Xing et al. [103] employed a two-step modification strategy to improve the thermal and pH stability of ZHD101 under acidic conditions. The initial step utilized Fireprot (http://loschmidt.chemi.muni.cz/fireprot) to predict mutation sites for enhanced thermal stability to obtain the mutant M1 containing seven amino acid mutations; the subsequent step predicted the relative burial of surface amino acids using the NetSurfP 3.0 server (https://services.healthtech.dtu.dk/services/NetSurfP-3.0/), leading to the mutant M2 by substituting K at the most exposed position with the polar, negatively charged D. As a result, the catalytic activity of M2 increased by 4.03-fold at 37.0 °C, pH 4.2. Furthermore, M2 demonstrated enhanced efficiency in hydrolyzing ZEN under conditions simulating the acidic environment of pig stomachs.

### Modifications based on enzymic similarity

The substitution of amino acid sequences within one of the structural domains of an enzyme, based on homology, represents a strategy to enhancing enzyme functionality. Jiang et al. [47] replaced the cap-structure domain of ZHD11B with that of ZHD518, resulting in a 1.5-fold, 1.6-fold, and 2.9-fold increase in the activity of Zhd11B-Zhd518 (130-170aa) against ZEN, α-ZAL, and β-ZAL, respectively (as shown in Table 7).

**Table 7:** Function proteins of ZEN-degrading enzymes

ZEN-degrading enzymes	Modifications	Functions	Notes	References
Zhd11B-Zhd518(130-170aa)	Structural domain replacement	Enhanced the catalytic activity towards ZEN, α-ZAL and β-ZAL by 1.5-, 1.6- and 2.9-fold	-	[47]
gfZHD101	Fusion protein	Located ZHD101 in *E. coli*, yeast, rice, and corn	Enhanced green fluorescent protein was fused to ZHD101	[16, 17, 20]
ZHD518	Short peptide	Enhanced protein expression by 1.28-fold, catalytic activity by 9.27-fold, thermal stability by 37.08-fold, and the stability for long-term storage	A multifunctional peptide S1v1-(AEAEAHAH)2 was fused to the N-terminus of ZHD518	[43]
Zhd11D	Short peptide	Enhanced catalytic activity by 1.5-fold and thermal stability at 40 °C by twofold	Amphiphilic short peptide S1 was fused to the N-terminus of Zhd11D	[105]
ZPF1	Fusion protein	Degraded AFB_1_ and ZEN simultaneously	ZEN hydrolase ZHD101.1 and Mn peroxidase Phcmnp were linked by the ligand peptide GGGGS	[106]
ZHDCP	Fusion protein	Degraded OTA and ZEN simultaneously	Combined the two single genes ZHD101 and carboxypeptidase in a frame deletion	[107]

Although there are fewer examples of such methods in ZEN-degrading enzymes, they have been successfully utilized in other enzymes. For instance, Yang et al. [108] enhanced the stability of the DON-degrading enzyme FHB7 using two homology-based approaches. Initially, a segment of amino acids (61–81) at the G site of FHB7 was replaced with a homologous sequence from *Gelatoporia subformispora*, thereby stabilizing the enzyme for purification and testing. Subsequently, the Consensus Finder tool (http://kazlab.umn.edu/) was employed to identify the conserved amino acid sites shared with the highly proteins similar to FHB7. Ten of these conserved sites were selected and validated, leading to the development of the mutant FHB7-M10, which exhibited a half-life 266.7-fold longer than that of the wild type [108].

### Modifications based on the fusion of additive components

Protein fusion expression is frequently employed to impart novel properties to ZEN-degrading enzymes. Depending on the specific additive used, these modifications can be mainly categorized as follows.

#### Modifications based on the fusion of fluorescent proteins

Fluorescent proteins are common components in protein fusion strategies, utilized for the localization of target proteins. In conventional methods [16, 17, 20], ZEN hydrolase ZHD101 was fused with enhanced green fluorescent protein to facilitate its localization within transgenic plants, *E. coli*, and yeast (as shown in Table 7).

In an innovative method [108], the fluorescent proteins were employed as a tool for screening correctly folded and stabilized proteins. Specifically, the “sandwich” structure of the DON-degrading enzyme FHB7, fused with the fluorescent protein CysG^A^, was utilized for high-throughput screening of mutants with enhanced stability, thereby significantly increasing the efficiency of screening benign random mutants. This method [108, 109] inserted the target protein into the two components of the fluorescent protein, forming a “sandwich” structure. When the target protein was correctly folded, the two parts of the fluorescent protein self-assembled and catalyzed the fluorescence; conversely, when the target protein structure was disrupted, the fluorescence disappeared. A notable advantage of this method is that thermal stability can be examined directly in engineered *E. coli* without protein purification. Additionally, it is applicable for high-throughput screening of random mutants of ZEN-degrading enzymes. Nonetheless, some of the high stability random mutants obtained by this method may exhibit reduced or lost original functionality [108], which necessitates further validation.

#### Modifications based on the fusion of short peptides

Incorporating short peptides into enzymes can facilitate proper folding, improve stability, and enhance the efficiency of heterologous expression. Wang et al. [43] fused an amphipathic short peptide S1 to the N-terminus of Zhd11D, resulting in both improved activity (1.5-fold) and thermostability (2-fold at 40 °C). Fang et al. [105] fused the multifunctional peptide S1v1-(AEAEAHAH)_2_ to the N-terminus of ZHD518 significantly enhanced protein expression by 1.28-fold, enzyme activity by 9.27-fold, and thermal stability by 37.08-fold after incubation at 45 °C for 10 min, in addition to enhancing enzyme stability during long-term storage. This improvement is attributed to the hydrophobic structure of short peptides, which promote oligomer formation, thereby enhancing the stability of enzymes. A comprehensive summary of these ZEN-degrading fusion enzymes was shown in Table 7.

In addition, more short peptides remain to be developed for ZEN-degrading enzymes. For example, the small ubiquitin-related modifier tag, a peptide with a molecular weight of 11.6 kDa, increased the production of recombinant proteins by increasing solubility and preventing degradation in the expression system [110–112]. Further, more short peptide tags (e.g., glutathione-S-transferase tag, maltose-binding protein tag and ubiquitin tag) may also improve the efficiency of heterologous expression of recombinant proteins [113, 114]. In the other hand, some short peptideshave been fused to enzymes to facilitate immobilization, including Spy chemistry [115], Leucine zipper [116, 117], SPFH domain [118, 119], split inteins GP41.1 [120], etc.

#### Modifications based on the fusion of function-independent proteins

An enzyme or structural domain with an independent function can be linked to a ZEN-degrading enzyme by a linker peptide to constitute a fusion protein. These function-independent structures were often other mycotoxin-degrading enzymes, thereby endowing the fusion protein with the ability to degrade multiple mycotoxins. Xia et al. [106] constructed the fusion enzyme ZPF1 by linking the ZEN hydrolase ZHD101.1 to the Mn peroxidase Phcmnp via the linker peptide GGGGS. ZPF1 was subsequently expressed in the food-grade strain *Kluyveromyces lactis* GG799 and verified in an optimized reaction system (including 1.0 mmol/L MnSO_4_, 0.1 mmol/L H_2_O_2_, 5.0 µg/mL AFB_1_, and 5.0 µg/mL ZEN, at 30 °C, 9 h), resulting in the degradation of AFB_1_ and ZEN by 64% and 46%, respectively. In an earlier study conducted by Azam et al. [107], the fusion protein ZHDCP, derived from the carboxypeptidase of *Bacillus amyloliquefaciens* ASAG and ZHD101, completely degraded 50 μmol/L ZEN at 2 h (35 °C, pH 7) and 50 μmol/L OTA at 30 min (37 °C, pH 7). A comprehensive summary of these ZEN-degrading fusion enzymes was shown in Table 7.

However, fusion proteins are more demanding in terms of induction and reaction conditions. The expression of the fusion enzyme ZPF1 by Xia et al. [106] was affected by the concentrations of galactose, MnSO_4_, and heme chloride. The optimal reaction conditions of the two enzymes fused were different, and the presence of Mn^2+^ and H_2_O_2_ might inhibit ZHD101.1. Consequently, the fusion enzyme ZPF1 exhibited lower degradation efficiencies for ZEN and AFB1 compared to the separate use of the two enzymes. Conversely, the attempt by Azam et al. [107] to fuse ZHD101 and carboxypeptidase was more effective, likely due to the greater similarity in the induction and reaction conditions required by the two enzymes.

### Modifications for the expression system of enzymes

Appropriate expression systems are critical for the heterologous expression of enzymes, as it influences the biological activity and recovery efficiency of the expressed enzymes, as well as their efficacy in practical applications.

#### Modifications for *Escherichia coli* expression system

The *E. coli* expression system is the most commonly utilized heterologous protein expression system in laboratory settings, primarily due to the extensive availability of commercially engineered strains. In numerous studies, *E. coli* BL21 (DE3) has been selected as the expression host for ZEN-degrading enzymes, often in conjunction with pET series plasmids, because of their maturity and reliability. Almost all ZHDs are able to be expressed and solubilized in this system, as well as bacterial laccases and peroxidases. However, laccases and peroxidases from fungi and plants may not fold correctly or exhibit biological activity in this expression system. Effective molecular chaperones may address this issue. For instance, Shao et al. [79] successfully expressed the heme peroxidase BaDyP from white-rot fungus *Bjerkandera adusta* using commercial *E. coli* BL21/pG-Tf2 (plasmid pG-Tf2 encoding three molecular chaperones groES, groEL, and tig) along with the low-temperature-inducible plasmid pCold I. Similarly, some researches [121, 122] have shown that fungal MnPs were difficult to be solubilized expression in *E. coli*, with molecular chaperones providing effective solutions. A comprehensive summary of these expression systems for ZEN-degrading enzymes was shown in Table 8.

**Table 8:** Modifications on the expression system of ZEN-degrading enzymes

Expression hosts	Expression vectors	Promoters	Signal peptides	Expression strategies	References
*Escherichia coli* BL21(DE3)	pET22b (+)	Lac	PgsA	Surface display	[48]
*Escherichia coli* BL21/pG-Tf2	pCold I	CSPA	-	Intracellular expression	[79]
*Escherichia coli* BL21(DE3)	pSB1C3	J23100	-	Surface display	[123]
*Pichia pastoris *GS115	pHBM905A	d1 + 2 × 201 AOX1 promoter	MF4I-SS	Secretory expression	[124]
*Pichia pastoris* X33	pPICZ(α)A	AOX1	α-Factor secretory signal	Secretory expression	[41]
*Pichia pastoris *GS115	pPIC9k	AOX1	Glucoamylase signal sequence	Secretory expression	[49]
*Pichia pastoris *GS115	pPIC9k	AOX1	α-Factor secretory signal	Secretory expression	[35, 63, 80]
*Saccharomyces cerevisiae* INVSc1	pYES2-alpha	GAL1	α-Factor secretory signal	Secretory expression	[125]
*Lactobacillus reuteri* Pg4	pNZ3004	LacA	LacA signal	Intracellular expression	[126]
*Lactobacillus reuteri* CGMCC 1.3264	pSlpA8148	P32	28 Signal peptides	Secretory expression and surface display	[127]
*Lactobacillus acidophilus *ATCC4356	pMG36e	P32	-	Intracellular expression	[89]
*Penicillium canescens* PCA-10	pNIC-Bsa4 and pXEG (PC1)	Inducible xylanase promoter	-	Secretory expression	[128]

#### Modifications for yeast expression system

Yeasts are well-established eukaryotic expression system. Commercial kits are straightforward and reliable tools. Yu et al. [41] utilized the *Pichia* Expression Kit to express ZEN hydrolase ZHD607. In addition, various methods have been employed to enhance the expression efficiency of ZEN-degrading enzymes in yeast, including strain optimization, promoter optimization [124], signal peptide optimization [49, 124], multicopying [124], etc. In instance, Xiang et al. [124] achieved efficient secretory expression of ZDH101 in *Pichia pastoris* GS115 by using the codon-optimized *ZHD101* gene, the optimized d1 + 2 × 201 AOX1 promoter, the MF4I-SS signal peptide, and the multi-copy expression cassette. Similarly, ZEN-degrading enzymes ZLHY-6 [49], ZENC [35], PpLac1 [63], AoLac2 [63], etc. have been expressed in *P. pastoris*. However, there are limited reports on the expression of ZEN-degrading enzymes in *Saccharomyces cerevisiae*. Tang et al. [125] expressed the peroxidase Prx from *Acinetobacter* sp. SM04 in *S. cerevisiae* INVSc1, observing a slightly lower effectiveness compared to the expression in *E. coli*, potentially due to the posttranslational modifications of Prx by *S. cerevisiae*. A comprehensive summary of these expression systems for ZEN-degrading enzymes was shown in Table 8.

Yeasts are extensively utilized as hosts for heterologous protein expression due to their capacity for achieving high expression levels, efficient secretion, post-translational modifications, and proper protein folding. However, a significant limitation is that secreted exogenous proteins may be truncated or incorrect glycosylation modifications. In addition, some yeasts have been reported to directly transform ZEN into α-ZOL, β-ZOL, or glycosylated ZEN, which remained highly estrogenic activity or could be re-transformed into ZEN [129, 130].

#### Modifications for *Lactobacillus* expression system

Probiotic additives are considered a promising strategy to combat the ZEN threat in feeds, thereby some ZEN-degrading enzymes have been heterologously expressed in *Lactobacillus*. Yang et al. [126] successfully expressed ZEN hydrolase ZHD101 in *L. reuteri.* This expression of heterologous ZHD101 did not adversely affect cell growth, acid and bile salt tolerance, and only minimally impacted the adhesion ability of *L. reuteri*. Similarly, Liu et al. [127] expressed RmZHD in *L. reuteri* by secretory expression and surface display. In another report [89], the ZEN oxidase Oxa was also successfully expressed in *L. acidophilus*. A comprehensive summary of these expression systems for ZEN-degrading enzymes was shown in Table 8.

The utilization of *Lactobacillus* for the expression of ZEN-degrading enzymes mainly aims to facilitate the degradation of ZEN within the animal digestive tract. However, comprehensive in vivo validation of these engineered probiotics remains limited. Some reports [131, 132] indicated that the absorption of ZEN in animal gut occurs rapidly and extensively, which may influence the efficacy of these engineered probiotics in degrading ZEN. In addition, the intestinal colonization of these engineered probiotics is also a challenge to be evaluated and resolved [133].

#### Modifications for other fungal expression systems

*Penicillium* and *Aspergillus* expression systems exhibit the potential to express ZEN-degrading enzymes. These systems are characterized by high growth rates, robust extracellular enzyme biosynthesis, straightforward and cost-effective medium compositions, and scalable fermentation processes. However, they are also associated with certain limitations, such as secreting endogenous proteases and producing secretions with high levels of endogenous heteroproteins. For *Penicillium*, Shcherbakova et al. [128] successfully achieved secretory expression of ZHD101 in *P. canescens* PCA-10, demonstrating in vitro degradation of ZEN (as shown in Table 8). Furthermore, many *Aspergillus* strains and their extracts have been reported to contain ZEN-degrading enzymes [31, 134, 135], suggesting potential industrial applications. Nonetheless, the expression of ZEN-degrading enzymes in *Aspergillus* has not yet been reported.

### Immobilization of ZEN-degrading enzymes

Immobilization plays a crucial role in enhancing the stability of enzymes and optimizing their applicability for specific scenarios. Here, we present a comprehensive overview of the documented strategies employed for the immobilization of ZEN-degrading enzymes.

#### Immobilization based on glutaraldehyde bridging agent

Using porous solid materials to adsorb or anchor ZEN-degrading enzymes is one of the traditional immobilization methods. Glutaraldehyde is commonly employed as a bridging agent in the immobilization of enzymes. He et al. [136] immobilized a crude enzyme solution of ZEN-degrading enzyme from *Aspergillus niger* FS10 onto treated rice husk, utilizing urea and glutaraldehyde as bridging agents. The immobilized crude enzyme solution demonstrated enhanced stability, increased resistance to heat and storage, and better performance in artificial digestive fluids. Similarly, Guo et al. [52] immobilized the ZEN-degrading laccase CotA onto chitosan microspheres (2–3 mm in diameter) using glutaraldehyde. This immobilization significantly improved the thermal stability of CotA, with its residual activity remaining above 87% compared to 34% for the free enzyme after heat treatment for 30 min at 80 °C. This method has also been applied to immobilize the ZEN-degrading enzymes rCotA [58], RhDypB [77], GhZH [36], etc.

Currently, this method is the most widely utilized for the immobilization of ZEN-degrading enzymes. Besides glutaraldehyde, other compounds such as glyoxal, epichlorohydrin, 1-ethyl-3-(3-dimethylaminopropyl) carbodiimide, and N-hydroxysuccinimide are frequently employed for cross-linking enzyme in immobilization [137] and could be considered for the immobilization of ZEN-degrading enzyme in the future.

#### Immobilization based on organic–inorganic hybrid nanoflowers

Organic–inorganic hybrid nanoflowers (HNFs) are hierarchical nanoparticles exhibiting a flower-like morphology, synthesized through a single bionanomorphic mineralization process [120]. This method offers advantages such as straightforward handling, mild reaction conditions, and no need for organic reagents. Zhou et al. [120] immobilized ZEN hydrolase ZHD518 onto Ca-P hybridized nanoflower crystals by split inteins GP41.1, and the immobilization of enzymes significantly improved its pH stability, specific activity, and reusability. Subsequently, the immobilized ZHD518 was evaluated in various application scenarios, including beer, beer wort, and edible oil, demonstrating effective ZEN degradation in each condition.

However, the preparation process of HNFs is time-consuming (usually requiring incubation for up to 3 d), which poses challenges for enzymes with low stability. In addition, direct treatment of the enzymes using the HNF method may result in over-embedding of the substrate channel of the enzyme, thereby impairing substrate-enzyme interactions and reducing catalytic efficiency [115]. Consequently, this method is frequently employed in conjunction with self-assembling enzyme components (e.g., Spy chemistry [115] and split inteins GP41.1 [120]).

#### Immobilization based on surface display

Surface display of enzymes on the outer wall of microbial cells has emerged as a novel immobilization strategy in recent years. Liu et al. [127] expressed the ZEN hydrolase RmZHD in *Lactobacillus reuteri* through surface display, enabling the engineered strain to completely hydrolyzed ZEN (2.5 mg/kg, 4 h) under low water conditions, as well as to effectively detoxify natural ZEN contamination in corn flour. Chen et al. [48] demonstrated the surface display of ZEN hydrolase ZHD-P in *E. coli* BL21 (DE3), resulting in enhanced pH stability of ZHD-P. In a separate study, Chen et al. [123] constructed a surface display system for ZEN hydrolase RmZHD in *E. coli* BL21 (DE3) using ice nucleation protein INPNC and the SpyTag/SpyCatcher system. The engineered *E. coli* degraded 94% of ZEN (1.898 μg/mL) within 1 h at 30 °C.

To date, the enzymes reported for surface display in ZEN degradation have exclusively been ZEN hydrolases. However, laccases and peroxidases have been successfully surface-displayed and utilized in other applications [138, 139], suggesting the potential for them in ZEN degradation. In addition, yeasts are also commonly used as hosts for surface display [140], yet there have no reports of displaying ZEN-degrading enzymes on the surface of yeasts. This represents a potential direction for future research.

#### Immobilization of enzymes in/on membranes

Immobilization of enzymes in/on membrane materials can reduce product inhibition, improve enzyme stability, increase the number of reaction cycles, and consistently separate the products from the biotransformation medium [141]. Dong et al. [142] covalently immobilized ZHD518 to tannic acid/3-(2-aminoethylamino) propyltriethoxysilane (TA/AEAPTES) and TA/AEAPTES/tetraethoxysilane-Fe^3+^ films. This immobilization significantly enhanced the reuse stability of the enzymes, maintaining 71% of their initial activity after eight cycles of reuse.

There are many methods for immobilizing enzymes in/on membranes, including embedding, adsorption, covalent bonding, and cross-linking [141]. These membranes are particularly effective in liquid treatments [141]. Although there is limited research for ZEN degradation through the enzymes in/on membrane materials, there have been reports of laccases and peroxidases being immobilized for the degradation of other hazardous compounds [143, 144]. It is anticipated that future developments will see more ZEN-degrading enzymes being immobilized using these methods.

#### Other methods of enzymic immobilization

Some methods [145] have not been employed for the immobilization of ZEN-degrading enzymes, but their outstanding functionality warrants attention, thereby they are briefly discussed here for potential inspiration.

Various strategies based on engineered strains have been employed to enzyme immobilization. For example, active inclusion bodies may serve as effective immobilization hosts for ZEN-degrading enzymes. Han et al. [116] co-localized four heterologous enzymes for the production of 1-butanol via fused leucine zippers to active inclusion bodies in *E. coli*, with this inclusion body scaffolded by a family II cellulose binding domain from *Cellulomonas fimi* exoglucanase. Xue et al. [117] utilized the same method to immobilize three heme-producing enzymes in *S. cerevisiae*. In addition, the active inclusion bodies themselves can improve the stability and catalytic efficiency of the immobilized enzymes [146–148]. Currently, many scaffolding proteins that can form active inclusion bodies have been reported, including SpyTag/Catcher chemistry [149], PhaC [150], SPFH [119], etc. Similarly, immobilization of ZEN-degrading enzymes into polymer/lipid inclusions, magnetosomes, and membrane vesicles in engineered microbes are also potential methods [145]. Moreover, spores of yeasts or some bacteria can also be immobilized hosts for enzyme encapsulation or surface display. These modified spores offer protection to enzymes under harsh conditions and improve the efficiency of multi-enzyme cascade reactions [151, 152].

The technologies for enzyme encapsulation are advancing rapidly and hold potential for the immobilization of ZEN-degrading enzymes, thereby enhancing their stability and utility. For example, molecular cages are a non-covalent binding method for enzyme immobilization, which can improve their stability and resistance to denaturants [153, 154], as well as improve the efficiency of multi-step enzymatic reactions [155]. Another prevalent method involves the use of metal–organic frameworks, which are extended porous network materials composed of metal-based nodes and organic connectors. These frameworks provide protection, enabling enzymes to remain active under adverse conditions such as denaturants, high temperatures, unnatural pH values, and organic solvents [156].

### Other improvement methods

Directed evolutionary mutagenesis based on random mutations can screen for enzymes with improved activity or stability. This method does not necessitate prior knowledge of the protein structure, while it requires screening thousands of colonies from mutation libraries, thereby necessitating a high-throughput screening method [157]. Although this method has not been directly applied to ZEN-degrading enzymes, it has been successfully employed in the optimization of laccases [157], peroxidases [158], and DON-degrading enzymes [108].

In summary, various modification strategies for ZEN-degrading enzymes exhibit unique focal points, and it is not appropriate to categorize any as superior or inferior. We propose that future approaches to modifying ZEN-degrading enzymes should integrate these strategies. For instance, one could enhance catalytic efficiency by mutating the active site of a natural ZEN-degrading enzyme, improve stability by altering non-active sites and fusing with short peptides, and further augment stability through immobilization.

## Applications for ZEN-degrading enzymes

The development of application scenarios that conform the physicochemical properties of ZEN-degrading enzymes is essential. Here, we delineate several applications of ZEN-degrading enzymes that have been reported in recent years.

### Enzyme degrade ZEN in vivo

The traditional application scenario of ZEN-degrading enzymes is adding them into feed and degrading ZEN in the digestion tract of animals. Gruber-Dorninger et al. assessed the efficacy of a commercial ZEN hydrolase ZENA in dairy cows (ZEN 10 mg/kg concentrate) [159], pigs (ZEN 200 µg/kg diet), chickens (ZEN 400 µg/kg diet), and rainbow trout (ZEN 2,000 µg/kg diet) [160]. ZENA effectively degraded ZEN in the digestive tract of these animals, yielding the degradation product HZEN. The application of ZENA resulted in a reduction of ZEN in the feces of pigs, chickens, and rainbow trout by over 50%. Dänicke et al. [161] further reported that ZENA degraded ZEN in the digestive tract of piglets, with the degradation product DHZEN in the feces, blood, and urine of the piglets. Additionally, ZENA significantly mitigated the ZEN-induced increase in uterine and ovarian weights. Song et al. [162] evaluated the effects of the commercial ZEN hydrolase Zymdetox Z-2000 on ZEN-challenged gilts (ZEN 0.4 mg/kg diet). The addition of Zymdetox Z-2000 or its coated variant resulted in a reduction of ZEN by 36.2%–42.3% in the stomach and 57.6%–73.6% in the duodenum. Until now, all ZEN-degrading enzymes reported in in vivo animal studies have been ZEN hydrolases, whereas the potential functions of laccases and peroxidases remain to be verified in the future.

The efficiency of ZEN-degrading enzymes in feeds or within animal body is influenced by some factors, including the types and properties of feeds (e.g., hot pelleting and wet mash), the species and digestive environments of animals (e.g., ruminants and birds), and the characteristics of the ZEN-degrading enzymes themselves. Although ZEN hydrolases exhibit high ZEN catalytic activity, they are limited by their relatively poor thermal stability and acid resistance, which may explain their reduced effectiveness of ZEN degradation in the complex digestive tract environment of animals.

To satisfy such application scenarios, future modifications to ZEN-degrading enzymes, informed by our experience, should focus on the following aspects: (1) Tolerance to temporary high temperatures (capable of withstanding temporary high temperatures during feed pelleting; e.g., 70 °C for 1–5 min). (2) High catalytic activity (capable of degrading over 60% of ZEN in digestive tract chyme within 1–6 h in vivo; e.g., ZEN 0.5 mg/kg feed). (3) Broad pH suitability (capable of degrading ZEN in gastric and intestinal juices; e.g., pH 2–8, 1–6 h). (4) High stability (maintains activity in stored feed; e.g., 30 d at 25 °C). (5) Tolerance to digestive enzymes (such as pepsin and trypsin).

### Enzyme degrade ZEN in vitro

The environments of the digestive tracts in animals are complex and uncontrollable, while the environments of the feed processing steps are more straightforward and manipulable. Consequently, the application scenarios of ZEN-degrading enzymes can be designed on this basis. Many reports have shown the effectiveness of ZEN-degrading enzymes to degrade ZEN in vitro, including feed samples [63], corn samples [84], peanut samples [80], corn flour [52, 58, 74], corn steep liquor [74], wheat flour [74], rice flour [74], etc. ZEN-degrading enzymes can function more efficiently under controlled artificial environments, especially laccases and peroxidases, because of the redox mediator they required.

Notably, the fusion of glucose oxidases (GODs) and peroxidases may help address the H₂O₂ limitation in the degradation of ZEN by peroxidases. GODs catalyze the oxidation of β-d-glucose to d-gluconic acid-δ-lactone and H₂O₂, utilizing molecular oxygen as the electron acceptor, thereby supplying the H₂O₂ required for the degradation of ZEN by peroxidases. Guo et al. [78] employed a dual-enzyme system combining GOD and dye-decolorizing peroxidase BsDyP to degrade ZEN (ZEN 10 μg/mL) in corn syrup, achieving a ZEN degradation rate of 33%.

Some other reports are more closely with the actual processes of industrial feed and feed ingredients production. André et al. [163] pretreated whole wheat kernels using cold-needle perforation and further incubated them with ZEN hydrolase ZHD518, resulting in a significant reduction of ZEN in both perforated and unperforated wheat kernels. Similarly, Chang et al. [49] used ZEN hydrolase ZLHY-6 to enzymatically remove ZEN from corn oil during alkaline refining process. The ZEN content was reduced from 617.45 to 13.00 µg/kg by neutralization and enzymatic detoxification (ZLHY-6 1.5 mg/mL, 40–45 °C, 2–5 h), achieving a ZEN degradation rate of 97.89%. Zhao et al. [164, 165] investigated the degradation of ZEN in corn oil by ZHD518, employing a similar method and optimized the process by response surface methodology.

In addition, other in vitro application scenarios warrant consideration, including fermented feed, production of feed ingredients in the agricultural by-product category, production of fruit pomace, alcoholic fermentation, etc. Some laccase [166], cellulases [166], and polysaccharidases [167] have been utilized in these application scenarios, suggesting the potential application scenarios of ZEN-degrading enzymes.

To satisfy such application scenarios, future modifications to ZEN-degrading enzymes, informed by our experience, should focus on the following aspects: (1) Tolerance to constant high temperatures (e.g., 40–60 °C for 24–72 h). (2) Tolerance to low pH levels (e.g., pH 3–5 for 24–72 h). (3) Applicability in organic solvents (e.g., corn oil and ethanol).

### Enzyme degrade ZEN in transgenic plants

The potential for expressing ZEN-degrading enzymes in plants to control ZEN accumulation at the source has been reported. Higa et al. [168] introduced ZEN hydrolase ZHD101 into a model monocotyledon rice plant to combat ZEN. ZHD101 was expressed and accumulated in five plants and was verified by Western Blot. Takahashi-Ando et al. [20] expressed ZHD101 in transgenic rice, the ZHD101 in calluses completely degraded ZEN in liquid media. Higa-Nishiyama et al. [16] reported that leaves of T1 offspring and seeds of T2 offspring from the rice transfected with ZHD101 effectively degraded ZEN. Igawa et al. [17] further transferred ZHD101 to transgenic maize, whose seeds efficiently degraded ZEN in the soaking solution at 20 and 28 °C.

Nevertheless, such methods have declined in popularity in recent years, likely due to concerns regarding the safety of genetically modified crops. On the other hand, the degradation of mycotoxins, which are metabolites of pathogenic microbes, may not be as critical for crops as the direct control of the pathogenic microbes themselves.

## Conclusions and future prospective

Currently, mycotoxin prevention and control are still an emerging scientific field, and the studies on the enzymatic detoxification of mycotoxins in feeds and feed ingredients, represented by ZEN-degrading enzymes, are growing rapidly. Nevertheless, the majority of mycotoxin-degrading enzymes are still confined to laboratory conditions and have yet to be widely adopted for industrial production. It is primarily due to some critical scientific challenges remain unresolved, including the toxicity assessment of mycotoxin degradation products, the simultaneous degradation of multiple mycotoxins, the inadequate enzyme stability and efficiency, etc. But regardless of the specific application scenario, the requirements for industrially applicable ZEN-degrading enzymes can be summarized as follows: (1) Safe degradation products. (2) High catalytic efficiency and stability. (3) Low life cycle cost.

Based on our personal experience, ZHDs represented by ZHD101 may be the closest ZEN-degrading enzymes to achieve this series of requirements. Their degradation products HZEN and DHZEN have received the relatively large number of safety validations; their catalytic activity and stability have also received the relatively large number of modifications for enhancement; and their industrial fermentation and practical applications are also the most numerous. However, the relatively poor thermal stability and acid resistance of ZHDs are still major limiting factors for their industrial applications. In our view, to obtain ZEN-degrading enzymes suitable for industrial production, the focus should be on further modifying existing ZEN-degrading enzymes. For instance, by leveraging big data analysis and artificial intelligence methods (e.g., protein consensus design) to redesign known ZHDs to enhance their catalytic efficiency and stability, and fused expressing them (e.g., incorporate short peptides) to enhance their stability and expression efficiency, ultimately immobilizing them to further enhance their stability (e.g., on yeast cell walls).

Overall, over the past two decades, a growing number of novel enzymes, new degradation mechanisms, and innovative enzymatic modification techniques have been discovered and applied. Particularly in recent years, advancements in big data analysis and artificial intelligence have facilitated research on ZEN-degrading enzymes, making solutions to these challenges seem within reach.

## Data Availability

Not applicable.

## Abbreviations

ABTS: 2-Azino-di-[3-ethylbenzo-thiazolin-sulphonate]
AFB_1_: Aflatoxin B_1_
DHZEN: Decarboxylated hydrolysed zearalenone
DON: Deoxynivalenol
ET: Electron transfer
GODs: Glucose oxidases
HAT: Hydrogen atom transfer
HNFs: Hybrid nanoflowers
HPLC: High Performance Liquid Chromatography
HZEN: Hydrolysed zearalenone
MnPs: Manganese peroxidases
OTA: Ochratoxin A
RMSF: Root Mean Square Fluctuation
SA: Syringaldehyde
TA/AEAPTES: Tannic acid/3-(2-aminoethylamino) propyltriethoxysilane
UHPLC-MS/MS: Ultra-High Performance Liquid Chromatography-Tandem Mass Spectrometry
ZAL: Zearalanol
ZEN: Zearalenone
ZHDs: The ZEN hydrolases of ZHD101 types
ZOLs: Zearalenols
